# Evaluation of Enoyl-Acyl Carrier Protein Reductase Inhibitors as *Pseudomonas aeruginosa* Quorum-Quenching Reagents

**DOI:** 10.3390/molecules15020780

**Published:** 2010-02-03

**Authors:** Liang Yang, Yang Liu, Claus Sternberg, Søren Molin

**Affiliations:** Department of Systems Biology, Technical University of Denmark, Kongens Lyngby, 2800, Denmark; E-Mails: yali@bio.dtu.dk (Y.L.); cst@bio.dtu.dk (C.S.); sm@bio.dtu.dk (S.M.)

**Keywords:** *Pseudomonas aeruginosa*, quorum-quenching, enoyl-acyl carrier protein reductase, molecular dynamics simulation

## Abstract

*Pseudomonas aeruginosa* is an opportunistic pathogen which is responsible for a wide range of infections. Production of virulence factors and biofilm formation by *P. aeruginosa* are partly regulated by cell-to-cell communication quorum-sensing systems. Identification of quorum-quenching reagents which block the quorum-sensing process can facilitate development of novel treatment strategies for *P. aeruginosa* infections. We have used molecular dynamics simulation and experimental studies to elucidate the efficiencies of two potential quorum-quenching reagents, triclosan and green tea epigallocatechin gallate (EGCG), which both function as inhibitors of the enoyl-acyl carrier protein (ACP) reductase (ENR) from the bacterial type II fatty acid synthesis pathway. Our studies suggest that EGCG has a higher binding affinity towards ENR of *P. aeruginosa* and is an efficient quorum-quenching reagent. EGCG treatment was further shown to be able to attenuate the production of virulence factors and biofilm formation of *P. aeruginosa*.

## 1. Introduction

*Pseudomonas aeruginosa* is an opportunistic pathogen which causes a wide range of infective diseases such as pulmonary infections, medical-device-related infections, urinary tract infections, wound infections as well as potentially fatal cystic fibrosis lung infections [1,2]. *P. aeruginosa* produces a large number of virulence factors and is notorious for its tolerance to many antimicrobial agents [3,4]. Another important feature of *P. aeruginosa* infections is the formation of surface attached complex multicellular communities, often referred to as biofilms [5,6]. Biofilm cells display multiple phenotypes and are surrounded by resistant extracellular polymeric substance (EPS) materials [7], which are often major causes for persistent infections [6]. Both production of virulence factors and biofilm formation are partly regulated by bacterial cell-to-cell communication (quorum-sensing) system in *P. aeruginosa* [3,8,9].

*Quorum sensing* (QS) is a widespread prokaryotic *intercellular communication system* which is based on the production of extracellular signal molecules (autoinducers) in relation to cell density [10]. Once the autoinducers reach their critical threshod concentrations, they can adjust the conformation of autoinducer receptors and together they can affect the expression profiles of a large number of genes [10]. There are three interconnected QS systems, *las*, *rhl* and *pqs* systems, in *P. aeruginosa* [3,11,12]. The major signal molecules involved in *las*, *rhl* and *pqs* QS systems are 3-oxo-C12-HSL, C4-HSL, and 2-heptyl-3-hydroxy-4-quinolone (PQS) respectively [12,13]. Among them, the *las* QS system is at the top of the QS hierarchy regulating the *rhl* and *pqs* QS systems [14].

Targeting pathways which are essential for the synthesis of QS molecules might be an approach for identifying quorum-quenching reagents. Recently, type II fatty acid synthesis intermediates were shown to be substrates for the LuxI family of autoinducer synthases [15]. The type II fatty acid synthesis pathway is present in most prokaryotes, plants, and several protozoans and has a different architectural organization from the type I fatty acid synthesis pathway of animals and human beings. In *P. aeruginosa*, mutations in the type II fatty acid synthesis *fabI* gene, which encodes enoyl-acyl carrier protein (ACP) reductase (ENR), lead to significant reduction of the 3-oxo-C12-HSL molecule of the *las* QS system [16]. High-throughput screening previously identified a large number of type II fatty acid synthesis inhibitors for different organisms [17]. These identified compounds have been served as scaffolds for structure based design of novel type II fatty acid synthesis inhibitors. Studying the effects of different type II fatty acid synthesis inhibitors on *P. aeruginosa* QS can provide valuable information for designing novel classes of *P. aeruginosa* quorum-quenching reagents.

In this study, we compared the effects of two reported broad spectrum type II fatty acid inhibitors, 5-chloro-2-(2,4-dichlorophenoxy)phenol (triclosan) and green tea (−)-epigallocatechin gallate (EGCG), on *P. aeruginosa* QS. These two inhibitors were reported be able to specifically bind with and inhibit the *Escherichia coli* ENR (EcENR) [18,19]. Since the complete three-dimensional structure of *P. aeruginosa* ENR (PaENR) is not available yet, we built a PaENR structure model through homology modeling. Then we used molecular dynamics (MD) simulations to analyze the binding affinities of triclosan and EGCG to PaENR. The MD results suggested that EGCG had a higher binding affinity to PaENR than triclosan. In agreement with the MD analysis, experiments showed that EGCG was a more efficient inhibitor of *P. aeruginosa* QS regulated virulence and biofilm formation than triclosan. 

## 2. Results and Discussion

The *P. aeruginosa* QS system is a model system for studies of the *N*-Acyl Homoserine Lactone (*AHLs*)-mediated QS in Gram-negative bacteria. The QS systems are widely used by pathogenic bacteria to coordinate expression of virulence products as well as biofilm formation, which cause a variety of persistent infections for human beings, animals and plants [20,21]. Recently, QS systems were proposed as a target for the development of anti-pathogenic drugs [22]. A number of quorum-quenching reagents were identified based on the structure of the QS signal molecules [22]. However, several of the QS molecule analogs are chemically unstable or toxic for animals and human beings [23,24,25]. Thus, alternative methods are needed to search for novel classes of quorum-quenching reagents with different structures from the QS molecules. In this work, we investigated a novel approach to identify quorum-quenching reagents through targeting the type II fatty acid synthesis pathway.

### 2.1. MD Simulation of Binding Affinities of Compounds to PaENR

**Figure 1:**
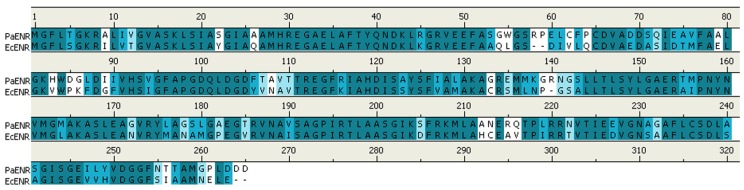
Pairwise protein sequence alignment of ENR from *P. aeruginosa* (PaENR) with ENR from *E. coli* (EcENR). Alignment was performed by Discovery Studio Visualizer 2.0 (Accelrys) and conserved residues are shown in dark blue with a white background.

The structure and functions of *E. coli* ENR (EcENR) are well studied by many different research groups. EcENR has high sequence similarity to the *P. aeruginosa* ENR (PaENR) based on blast searching of PaENR sequence in the RCSB protein data bank [26]. Pairwise protein sequence alignment shows that the essential amino acids of EcENR which are involved in binding of NADH to EcENR [27] are conserved between these two ENRs: the residues in PaENR corresponding to Gly-93, Met-159 and Phe-203 of EcENR are Gly-95, Met-162 and Phe-206 (Figure 1).

**Figure 2:**
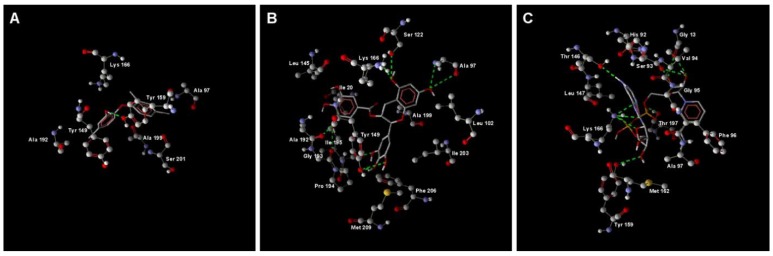
Hydrogen bond interaction analysis by Molegro Molecular Viewer. A: PaENR-triclosan; B: PaENR-EGCG; C: PaENR-NADH.

We have used the EcENR structures to build a *P. aeruginosa* ENR (PaENR) model by homology modeling with Modeller. Then we used molecular dynamics simulation to investigate the binding affinities of two reported EcENR inhibitors, triclosan and EGCG, to PaENR. Since bacterial ENR activity is *NADH* dependent, and both triclosan and EGCG were reported to competitively bind with ENR against NADH [18,19], we also simulated the binding affinity and mode of NADH with PaENR. The RMSD values of all of the three systems became stable after 2.4 ns simulation (data not shown). After MD simulation, we examined the final structures of the PaENR-triclosan, PaENR-EGCG and PaENR-NADH complexes by binding score analysis and hydrogen bond interaction analysis with Molegro Molecular Viewer. The estimated binding scores of triclosan, EGCG and NADH to PaENR were -84.9197, -157.744 and -194.123 respectively and the hydrogen bond interactions of triclosan, EGCG and NADH to PaENR are shown in Figure 2. Based on this analysis, triclosan has fewer hydrogen bond interactions with PaENR than EGCG and NADH. The hydroxyl groups from EGCG enable it to form several hydrogen bonds with PaENR (Figure 2B). Most of the hydrogen bond interactions of PaENR-triclosan, PaENR-EGCG and PaENR-NADH complexes are mediated by amino acid residues close to residue 95, residue 150 and residue 200 (Figure 2), which is in accordance with the experimental crystal structure analysis of ENRs from other species [27]. Based on the MD simulation analysis, EGCG has a higher binding affinity to PaENR than triclosan and this result suggests that EGCG may have better inhibition effects towards QS activity via interfering PaENR than triclosan. By employing another approach, Sharma *et al*. used cluster analysis to group docked confirmations generated through 100 independent docking runs of triclosan and EGCG against Enoyl-ACP Reductase from Plasmodium falciparum (PfENR) [28]. Similar to our results, they reported that triclosan and EGCG tend to occupy different binding pockets of the PfENR. Our method using Molegro Molecular Viewer to calculate the binding affinities of triclosan, EGCG and NADH to PaENR is based on simple energy functions, which is fast but provides relatively low accuracy. Rigorous free energy perturbation methods after MD simulation could obtain more accurate protein–ligand binding energy [29].

### 2.2. Quorum-Sensing, Virulence and Biofilm Attenuation by PaENR Inhibitors

It was shown previously that a high binding affinity to EcENR is very critical for EcENR inhibitors to act as antibiotics, since the AcrAB efflux system of *E. coli* can efficiently pump EcENR inhibitors out of the cells [30]. *P. aeruginosa* has an efflux system highly homologous to the *E. coli* AcrAB system, which was shown to be essential for its resistance to the ENR inhibitor triclosan [16]. Triclosan is, however, not an efficient PaENR inhibitor, and it is actually widely used in the *Pseudomonas isolation agar* (PIA; Difco) (whose formulation contains 25 μg/mL of *triclosan*) to isolate *Pseudomonas* species from other bacterial species (such as *E. coli*). We propose here that a high binding affinity of compounds to PaENR is necessary for them to efficiently interfere with the *P. aeruginosa* type II fatty acid synthesis and attenuate QS signaling.

**Figure 3:**
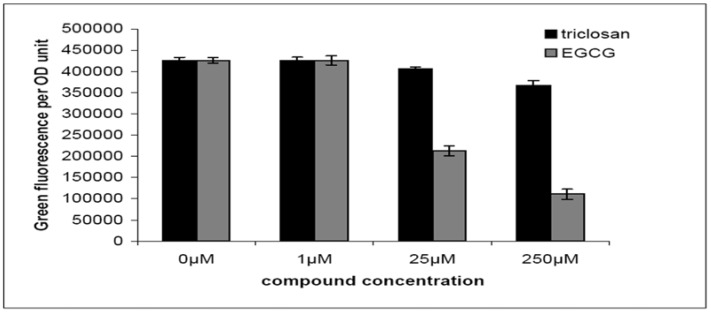
Expression of *lasB*:*gfp*(*ASV*) in wild-type *P*. *aeruginosa* treated with triclosan (black bar) and EGCG (gray bar). Results are average values of green fluorescence divided by OD_600_ taken from a single time point measurement corresponding to maximal induction of the reporters in the late log phase of growth. Inhibitors were added at concentrations of 0 µM, 1 µM, 25 µM and 250 µM. Averages and SDs of three replicates are shown.

To test our proposal we examined the quorum-quenching capabilities of triclosan and EGCG by means of a quorum-quenching assay based on a *lasB*::*gfp(ASV)* translational fusion in *P*. *aeruginosa* wild-type PAO1 strain [31]. In this assay an unstable version of Gfp (ASV) was fused with the QS regulated LasB protein, by which the *P. aeruginosa* QS activity can be indicated by measuring green fluorescence per OD_600_ unit. As shown in Figure 3, triclosan showed a slight reduction in green fluorescence per OD unit, while EGCG showed significant reduction in green fluorescence per OD unit. Both triclosan and EGCG showed no growth inhibition of *P*. *aeruginosa* PAO1 strain under our tested concentrations. This suggests that EGCG is an active quorum-quenching reagent.

We also investigated whether attenuation of *P. aeruginosa* QS by EGCG might lead to reduction of QS regulated virulence factors and activities *via* measuring transcription of *pqsABCDE* operon and swarming motility of *P. aeruginosa* PAO1 strain. 

**Figure 4:**
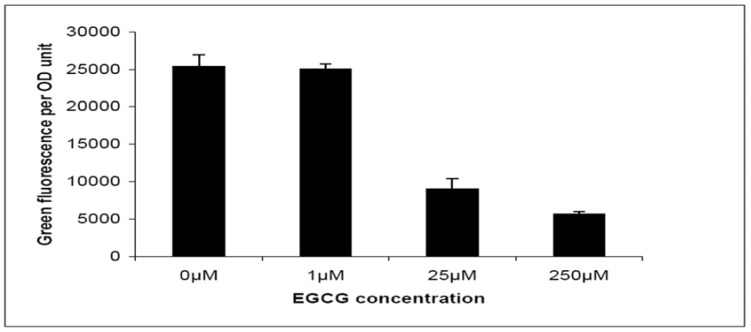
Expression of *pqsA*:*gfp*(*ASV*) in wild-type *P*. *aeruginosa* treated with EGCG. Results are average values of green fluorescence divided by OD_600_ taken from a single time point measurement corresponding to maximal induction of the reporters in the late log phase of growth. Inhibitors were added at concentrations of 0 µM, 1 µM, 25 µM and 250 µM. Averages and SDs of three replicates are shown.

The *pqsABCDE* operon is involved in synthesis of *Pseudomonas* quinolone signal (*PQS*), which functions as a signal molecule as well as an iron chelator [32]. The transcription of the *pqsABCDE* operon was shown to be under stringent regulation of the *las* QS system [33]. In the *pqsABCDE* transcription assay, an unstable version of Gfp (ASV) was fused with a QS regulated *pqsABCDE* promoter, by which the transcription of the *pqsABCDE* operon can be estimated by measuring green fluorescence per OD unit [34]. As shown in Figure 4, transcription of the *pqsABCDE* operon in the *P. aeruginosa* PAO1 was reduced by EGCG in a dose dependent manner. The swarming motility is characterized by a coordinated translocation of a bacterial population across solid or semi-solid surfaces. By dropping a cell pellet on the surface of a solid agar plate, bacterial cells are able to move away from the initial location leading to the formation of arm-like or dendritic fractal-like patterns. 

**Figure 5:**
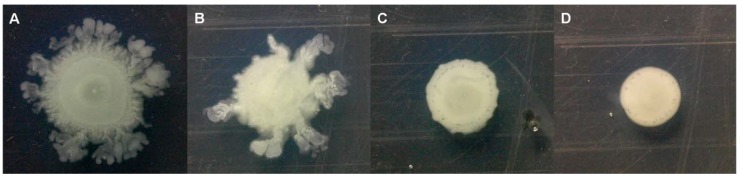
Swarming motility of wild-type *P. aeruginosa* strain on agar plates containing 0 µM (A), 1 µM (B), 25 µM (C) and 250 µM EGCG (D).

Since it was reported that both type II fatty acid synthesis [35] and PQS regulation [36] were involved in production of the biosurfactant rhamnolipid, which is required for *P. aeruginosa* swarming motility, we investigated whether EGCG could attenuate swarming motility of *P. aeruginosa* PAO1 strain. As expected, our results showed that swarming motility by *P. aeruginosa* PAO1 strain was reduced by EGCG in a dose dependent manner (Figure 5). 

**Figure 6:**
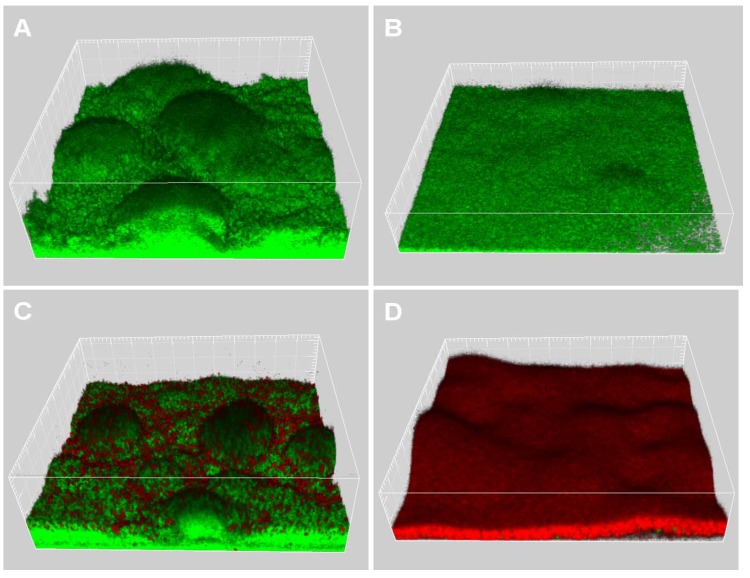
4-days-old biofilms of Gfp-tagged wild-type grown in FAB medium without (A) or with 25 µM EGCG (B). Biofilms were further treated with 50 µg/ml ciprofloxacin for 24 h (C: biofilm grown in FAB without EGCG; D: biofilm grown in FAB with 25 µM EGCG), after which they were stained with propidium iodide and images were acquired by CLSM. Live cells appear green and dead cells appear red.

*P. aeruginosa* QS and swarming motility have been shown to play important roles in antibiotic resistance and biofilm formation [37,38,39]. Thus we further investigated the effects of EGCG on *P. aeruginosa* biofilm formation and its antibiotic resistance. In order to examine the effects of EGCG on *P. aeruginosa* biofilm formation, we grew Gfp tagged *P. aeruginosa* PAO1 in flow-chambers irrigated with normal FAB medium, with or without 25 µM EGCG. After 4 days of growth, the biofilms were observed by confocal laser scanning microscopy (CLSM), which can acquire in-focus images from selected depths based on fluorescence of the samples. Biofilm images were acquired point-by-point and three-dimensional structures of biofilms were reconstructed using the IMARIS software package (Bitplane AG). The *P. aeruginosa* PAO1 grown in normal FAB medium without EGCG formed heterogeneous biofilms containing mushroom-shaped multicellular structures (Figure 6A), while *P. aeruginosa* PAO1 grown in FAB medium with 25 µM EGCG formed biofilms displaying less spatially organized multicellular structures (Figure 6B). After 4 days of growth, the biofilms were treated with 50 µg/mL ciprofloxacin for 24 hours. The dead bacteria in the biofilms were visualized by CLSM after staining with propidium iodide. The results showed that ciprofloxacin treatment caused killing of the bacteria located in the outer part of the cap-portion of the mushroom-shaped structures in 4-days-old *P. aeruginosa* PAO1 biofilms grown in normal FAB medium (Figure 6C), while ciprofloxacin treatment caused killing of most of bacteria in *P. aeruginosa* PAO1 biofilms grown in FAB medium with 25 µM EGCG (Figure 6D). These results are in accordance with previous studies showing that quorum-quenching reagent treated biofilms are more sensitive to treatments by antimicrobial agents [34,40,41].

## 3. Experimental

### 3.1. Homology Modeling

The sequence of the PaENR protein is highly homologous to the EcENR protein (sequence similarity = 84.0%, sequence identity = 68.8%). We have built a three-dimensional structure model of PaENR based on the 1.75 Å crystal structure of EcENR (accession code: 1QSG) [42]. Homology modeling was performed with program Modeller 9V6 [43]. The model was assessed by VARIFY 3D [44,45]. Out of the few predicted iterative models, the best model having the lowest value of the MODELLER objective function was selected for the further analysis. The 3D structures of triclosan, EGCG and NADH ligands were obtained from the PDBeChem database [46]. The ligands were then inserted into the PaENR with Molegro Molecular Viewer v1.2.0 (http://www.molegro.com/) and saved as protein-ligand complexes respectively.

### 3.2. Molecular Dynamics Simulations

The molecular dynamics (MD) package Desmond 2.2 (D.E. Shaw Research & Schrödinger) and OPLS-AA 2005 force field [47] were used for MD simulations of the three computationally built PaENR-ligand complexes. Hydrogen atoms were added using the protein preparation wizard in Maestro 9.0 (Schrödinger). Each structure was embedded in a box of TIP3P water molecules [48] with the buffering distance set to 10 Å. For long range electrostatics, the smooth particle mesh Ewald method [49] was used along with periodic boundary conditions. By assuming normal charge states of ionizable groups corresponding to pH 7, sodium (Na^+^) and chloride (Clˉ) counter-ions at physiological concentration of 0.015 mol/L were added in the box in random positions to ensure the global charge neutrality. The starting structures were relaxed by performing minimization of the model system using a hybrid method of the steepest descent and the limited-memory Broyden-Fletcher-Goldfarb-Shanno (LBFGS) algorithms [50] with a convergence threshold for the gradient in units of 1.0 kcal Å/mol. The minimization was followed by equilibration dynamics at constant temperature (T 310K) and constant pressure (P 1 bar). 2.4 ns of production MD simulations were performed for each PaENR-ligand complex with an integration time step of 2 fs. The constant pressure and temperature were controlled via Langevin dynamics method [51]. Snapshot structures were extracted for every 0.6 ps, resulting in 4000 structures from each trajectory. Thermodynamic properties (temperature, pressure, volume and energy) were monitored during MD simulations to check their convergence to stable values. Analysis of the trajectories was performed using the VMD 1.8.7 [52]. The hydrogen bond interactions and binding scores of PaENR-ligand complexes were analyzed by using Molegro Molecular Viewer.

### 3.3. Bacterial Cell Based Quorum-Quenching Assay 

5-Chloro-2-(2,4-dichlorophenoxy)phenol (triclosan; ≥97.0%) and (−)-epigallocatechin gallate (EGCG; ≥ 97.0%) were purchased from Sigma-Aldrich. The quorum-quenching assay was carried out by growing the *P*. *aeruginosa lasB*::*gfp (ASV)* QS reporter strain [30] in a 96-well microtiter tray (black polystyrene; Nunc) in AB medium [53] together with 0 µM, 1 µM, 25 µM and 250 µM of triclosan and EGCG. The microtiter tray was incubated at 34 °C over night without shaking and then measured in a Wallac 1420 VICTOR3 plate reader (Perkin-Elmer, MA). The instrument was set to measure green fluorescent protein (Gfp) expression by means of the protein’s fluorescence at 535 nm upon excitation at 485 nm and the growth in the wells as the OD_600_. 

### 3.4. Virulence Inhibition Assays 

PQS synthesis assay and swarming motility assay were used as previously reported [54] with modification to measure the production of QS regulated virulence factors. For the PQS synthesis assay, the expression of a plasmid-borne *pqsA*::*gfp(ASV)* fusion in the microtitre tray cultures of the PAO1 wild-type grown in AB medium with 0 µM, 1 µM, 25 µM and 250 µM of EGCG was measured in the same manner as the above *P*. *aeruginosa lasB*::*gfp* QS reporter strain quorum-quenching assay. For the swarming motility assay, swarming plates consisted of AB minimal medium supplemented with glucose, casamino acids, and 0.5% Bacto agar. EGCG was mixed into the medium immediately before casting at concentrations of 0 µM, 1 µM, 25 µM and 250 µM. The plates were left for drying without lids in a fume hood for 1 h at room temperature. Five-microliter drops of overnight cultures of *P. aeruginosa* wild-type PAO1 strain were placed on the prepared plates and incubated for 18 hours at room temperature before pictures of each plate were taken.

### 3.5. Biofilm Assay 

Biofilms were grown for 4 days in flow chambers with individual channel dimensions of 1 × 4 × 40 mm. The flow system was assembled and prepared as described previously [55]. Inoculation of the system was carried out by injecting 300 µL 1:1,000 diluted over night culture of a Gfp tagged *P. aeruginosa* strain [34] into flow channel with a small syringe. After inoculation, the flow chambers were left upside down without flow for 1 h to allow bacterial attachment to the glass cover, after which medium flow was started with a Watson Marlow 205S peristaltic pump. The flow chambers were irrigated with medium with or without 25 µM EGCG. The mean flow velocity in the flow chambers was 0.2 mm s/L, corresponding to laminar flow with a Reynolds number of 0.02. The biofilms were grown at 30 °C. Biofilm tolerance to ciprofloxacin was assessed by irrigating 4-day-old flow-chamber-grown *P. aeruginosa* biofilms with medium containing 50 µg/mL ciprofloxacin (MIC = 1 µg/mL) for 24 h, followed by staining of the dead cells with propidium iodide, and CLSM image acquisition. All microscopy observations and image acquisitions were done with a Zeiss LSM510 confocal laser scanning microscope (CLSM) equipped with detectors and filter sets for monitoring of Gfp and propidium iodide fluorescence. Images were obtained using a 40x/1.3 objective. Simulated three-dimensional images and sections were generated using the IMARIS software package (Bitplane AG).

## 4. Conclusions 

Our study suggests that the type II fatty acid synthesis pathway is an alternative target for development of anti-virulence drug lead compounds. Type II fatty acid synthesis pathway inhibitors can be used synergistically with conventional antibiotics to treat bacterial biofilm related infections. Our study also shows that molecular dynamics simulation can be used as a tool to predict the binding mode and the affinity of compounds to target proteins in a relatively accurate manner. Future work of screening novel type II fatty acid synthesis pathway inhibitors could be done by molecular docking based virtual screening followed by molecular dynamics simulations.

## References

[1] Bodey G.P., Bolivar R., Fainstein V., Jadeja L. (1983). Infections caused by Pseudomonas aeruginosa. Rev. Infect Dis..

[2] Gibson R.L., Burns J.L., Ramsey B.W. (2003). Pathophysiology and management of pulmonary infections in cystic fibrosis. Am. J. Respir. Crit. Care Med..

[3] Passador L., Cook J.M., Gambello M.J., Rust L., Iglewski B.H. (1993). Expression of Pseudomonas aeruginosa virulence genes requires cell-to-cell communication. Science.

[4] Carmeli Y., Troillet N., Karchmer A.W., Samore M.H. (1999). Health and economic outcomes of antibiotic resistance in Pseudomonas aeruginosa. Arch. Intern. Med..

[5] Hoiby N., Krogh Johansen H., Moser C., Song Z., Ciofu O., Kharazmi A. (2001). Pseudomonas aeruginosa and the *in vitro* and *in vivo* biofilm mode of growth. Microbes Infect..

[6] Costerton J.W., Stewart P.S., Greenberg E.P. (1999). Bacterial biofilms: A common cause of persistent infections. Science.

[7] Sauer K., Camper A.K., Ehrlich G.D., Costerton J.W., Davies D.G. (2002). Pseudomonas aeruginosa displays multiple phenotypes during development as a biofilm. J. Bacteriol..

[8] Parsek M.R., Greenberg E.P. (1999). Quorum sensing signals in development of Pseudomonas aeruginosa biofilms. Meth. Enzymol..

[9] Kjelleberg S., Molin S. (2002). Is there a role for quorum sensing signals in bacterial biofilms?. Curr. Opin. Microbiol..

[10] Williams P., Camara M., Hardman A., Swift S., Milton D., Hope V.J., Winzer K., Middleton B., Pritchard D.I., Bycroft B.W. (2000). Quorum sensing and the population-dependent control of virulence. Philos. Trans. R. Soc. Lond., B, Biol. Sci..

[11] Latifi A., Winson M.K., Foglino M., Bycroft B.W., Stewart G.S., Lazdunski A., Williams P. (1995). Multiple homologues of LuxR and LuxI control expression of virulence determinants and secondary metabolites through quorum sensing in Pseudomonas aeruginosa PAO1. Mol. Microbiol..

[12] Pesci E.C., Milbank J.B., Pearson J.P., McKnight S., Kende A.S., Greenberg E.P., Iglewski B.H. (1999). Quinolone signaling in the cell-to-cell communication system of Pseudomonas aeruginosa. Proc. Natl. Acad. Sci. USA.

[13] Latifi A., Foglino M., Tanaka K., Williams P., Lazdunski A. (1996). A hierarchical quorum-sensing cascade in Pseudomonas aeruginosa links the transcriptional activators LasR and RhIR (VsmR) to expression of the stationary-phase sigma factor RpoS. Mol. Microbiol..

[14] Wagner V.E., Bushnell D., Passador L., Brooks A.I., Iglewski B.H. (2003). Microarray analysis of Pseudomonas aeruginosa quorum-sensing regulons: Effects of growth phase and environment. J. Bacteriol..

[15] Val D.L., Cronan J.E. (1998). *In vivo* evidence that S-adenosylmethionine and fatty acid synthesis intermediates are the substrates for the LuxI family of autoinducer synthases. J. Bacteriol..

[16] Hoang T.T., Schweizer H.P. (1999). Characterization of Pseudomonas aeruginosa enoyl-acyl carrier protein reductase (FabI): A target for the antimicrobial triclosan and its role in acylated homoserine lactone synthesis. J. Bacteriol..

[17] Campbell J.W., Cronan J.E. (2001). Bacterial fatty acid biosynthesis: Targets for antibacterial drug discovery. Annu. Rev. Microbiol..

[18] Heath R.J., Rubin J.R., Holland D.R., Zhang E., Snow M.E., Rock C.O. (1999). Mechanism of triclosan inhibition of bacterial fatty acid synthesis. J. Biol. Chem..

[19] Zhang Y.M., Rock C.O. (2004). Evaluation of epigallocatechin gallate and related plant polyphenols as inhibitors of the FabG and FabI reductases of bacterial type II fatty-acid synthase. J. Biol. Chem..

[20] Singh P.K., Schaefer A.L., Parsek M.R., Moninger T.O., Welsh M.J., Greenberg E.P. (2000). Quorum-sensing signals indicate that cystic fibrosis lungs are infected with bacterial biofilms. Nature.

[21] De Kievit T.R., Iglewski B.H. (2000). Bacterial quorum sensing in pathogenic relationships. Infect. Immun..

[22] Rasmussen T.B., Givskov M. (2006). Quorum-sensing inhibitors as anti-pathogenic drugs. Int. J. Med. Microbiol..

[23] Glansdorp F.G., Thomas G.L., Lee J.K., Dutton J.M., Salmond G.P., Welch M., Spring D.R. (2004). Synthesis and stability of small molecule probes for Pseudomonas aeruginosa quorum sensing modulation. Org. Biomol. Chem..

[24] Hentzer M., Givskov M. (2003). Pharmacological inhibition of quorum sensing for the treatment of chronic bacterial infections. J. Clin. Invest.

[25] Telford G., Wheeler D., Williams P., Tomkins P.T., Appleby P., Sewell H., Stewart G.S., Bycroft B.W., Pritchard D.I. (1998). The Pseudomonas aeruginosa quorum-sensing signal molecule *N*-(3-oxododecanoyl)-L-homoserine lactone has immunomodulatory activity. Infect. Immun..

[26] Deshpande N., Addess K.J., Bluhm W.F., Merino-Ott J.C., Townsend-Merino W., Zhang Q., Knezevich C., Xie L., Chen L., Feng Z., Green R.K., Flippen-Anderson J.L., Westbrook J., Berman H.M., Bourne P.E. (2005). The RCSB Protein Data Bank: A redesigned query system and relational database based on the mmCIF schema. Nucleic Acids Res..

[27] Baldock C., Rafferty J.B., Stuitje A.R., Slabas A.R., Rice D.W. (1998). The X-ray structure of Escherichia coli enoyl reductase with bound NAD+ at 2.1 A resolution. J. Mol. Biol..

[28] Kumar S.S., Prasanna P., Gyanendra K., Namita S., Avadhesha S. (2007). Green tea catechins potentiate triclosan binding to enoyl-ACP reductase from Plasmodium falciparum (PfENR). J. Med. Chem..

[29] Woo H.J. (2008). Calculation of absolute protein-ligand binding constants with the molecular dynamics free energy perturbation method. Methods Mol. Biol..

[30] Chuanchuen R., Beinlich K., Hoang T.T., Becher A., Karkhoff-Schweizer R.R., Schweizer H.P. (2001). Cross-resistance between triclosan and antibiotics in Pseudomonas aeruginosa is mediated by multidrug efflux pumps: Exposure of a susceptible mutant strain to triclosan selects nfxB mutants overexpressing MexCD-OprJ. Antimicrob. Agents Chemother. (Bethesda).

[31] Rasmussen T.B., Bjarnsholt T., Skindersoe M.E., Hentzer M., Kristoffersen P., Kote M., Nielsen J., Eberl L., Givskov M. (2005). Screening for quorum-sensing inhibitors (QSI) by use of a novel genetic system, the QSI selector. J. Bacteriol..

[32] Bredenbruch F., Geffers R., Nimtz M., Buer J., Haussler S. (2006). The Pseudomonas aeruginosa quinolone signal (PQS) has an iron-chelating activity. Environ. Microbiol..

[33] McGrath S., Wade D.S., Pesci E.C. (2004). Dueling quorum sensing systems in Pseudomonas aeruginosa control the production of the Pseudomonas quinolone signal (PQS). FEMS Microbiol. Lett..

[34] Yang L., Barken K.B., Skindersoe M.E., Christensen A.B., Givskov M., Tolker-Nielsen T. (2007). Effects of iron on DNA release and biofilm development by Pseudomonas aeruginosa. Microbiology.

[35] Rehm B.H., Mitsky T.A., Steinbuchel A. (2001). Role of fatty acid de novo biosynthesis in polyhydroxyalkanoic acid (PHA) and rhamnolipid synthesis by pseudomonads: Establishment of the transacylase (PhaG)-mediated pathway for PHA biosynthesis in Escherichia coli. Appl. Environ. Microbiol..

[36] Diggle S.P., Winzer K., Chhabra S.R., Worrall K.E., Camara M., Williams P. (2003). The Pseudomonas aeruginosa quinolone signal molecule overcomes the cell density-dependency of the quorum sensing hierarchy, regulates rhl-dependent genes at the onset of stationary phase and can be produced in the absence of LasR. Mol. Microbiol..

[37] Overhage J., Bains M., Brazas M.D., Hancock R.E. (2008). Swarming of Pseudomonas aeruginosa is a complex adaptation leading to increased production of virulence factors and antibiotic resistance. J. Bacteriol..

[38] Shrout J.D., Chopp D.L., Just C.L., Hentzer M., Givskov M., Parsek M.R. (2006). The impact of quorum sensing and swarming motility on Pseudomonas aeruginosa biofilm formation is nutritionally conditional. Mol. Microbiol..

[39] Davies D.G., Parsek M.R., Pearson J.P., Iglewski B.H., Costerton J.W., Greenberg E.P. (1998). The involvement of cell-to-cell signals in the development of a bacterial biofilm. Science.

[40] Bjarnsholt T., Jensen P.O., Burmolle M., Hentzer M., Haagensen J.A., Hougen H.P., Calum H., Madsen K.G., Moser C., Molin S., Hoiby N., Givskov M. (2005). Pseudomonas aeruginosa tolerance to tobramycin, hydrogen peroxide and polymorphonuclear leukocytes is quorum-sensing dependent. Microbiology.

[41] Hentzer M., Wu H., Andersen J.B., Riedel K., Rasmussen T.B., Bagge N., Kumar N., Schembri M.A., Song Z., Kristoffersen P., Manefield M., Costerton J.W., Molin S., Eberl L., Steinberg P., Kjelleberg S., Hoiby N., Givskov M. (2003). Attenuation of Pseudomonas aeruginosa virulence by quorum sensing inhibitors. EMBO J..

[42] Stewart M.J., Parikh S., Xiao G., Tonge P.J., Kisker C. (1999). Structural basis and mechanism of enoyl reductase inhibition by triclosan. J. Mol. Biol..

[43] Eswar N., Eramian D., Webb B., Shen M.Y., Sali A. (2008). Protein structure modeling with MODELLER. Methods Mol. Biol..

[44] Bowie J.U., Luthy R., Eisenberg D. (1991). A method to identify protein sequences that fold into a known three-dimensional structure. Science.

[45] Luthy R., Bowie J.U., Eisenberg D. (1992). Assessment of protein models with three-dimensional profiles. Nature.

[46] Dimitropoulos D., Ionides J., Henrick K. (2006). Using MSDchem to search the PDB ligand dictionary. Curr. Protoc. Bioinform..

[47] Jorgensen W.L., Maxwell D.S., Tirado-Rives J. (1996). Development and testing of the opls all-atom force field on conformational energetics and properties of organic liquids. J. Am. Chem. Soc..

[48] Jorgensen W.L., Chandrasekhar J., Madura J.D., Impey R.W., Klein M.L. (1983). Comparison of simple potential functions for simulating liquid water. J. Chem. Phys..

[49] Essmann U., Perera L., Berkowitz M.L. (1995). A smooth particle mesh Ewald method. J. Chem. Phys..

[50] Broyden C.G. (1970). The convergence of a class of double-rank minimization algorithms. IMA J. Math. Appl..

[51] Paterlini M.G., Ferguson D.M. (1998). Constant temperature simulations using the Langevin equation with velocity Verlet integration. Chem. Phys..

[52] Humphrey W., Dalke A., Schulten K. (1996). VMD: Visual molecular dynamics. J. Mol. Graph..

[53] Clark D.J., Maaløe O. (1967). DNA replication and the division cycle in Escherichia coli. J. Mol. Biol..

[54] Yang L., Rybtke M.T., Jakobsen T.H., Hentzer M., Bjarnsholt T., Givskov M., Tolker-Nielsen T. (2009). Computer-aided identification of recognized drugs as Pseudomonas aeruginosa quorum-sensing inhibitors. Antimicrob. Agents Chemother. (Bethesda).

[55] Sternberg C., Tolker-Nielsen T. (2006). Growing and analyzing biofilms in flow cells. Curr. Protoc. Microbiol..

